# The Level of Vitamin D in Children and Adolescents with Nonalcoholic Fatty Liver Disease: A Meta-Analysis

**DOI:** 10.1155/2019/7643542

**Published:** 2019-07-14

**Authors:** Shanshan Zhu, Yuhui Wang, Fei Luo, Jie Liu, Liangchang Xiu, Jiheng Qin, Tao Wang, Na Yu, Hongfu Wu, Tangbin Zou

**Affiliations:** ^1^Dongguan Key Laboratory of Environmental Medicine, School of Public Health, Guangdong Medical University, Dongguan 523808, China; ^2^The Third Affiliated Hospital of Guangdong Medical University (Longjiang Hospital of Shunde District), Foshan 528318, China; ^3^Dongguan Scientific Research Center, Guangdong Medical University, Dongguan 523808, China; ^4^School of Basic Medical Sciences, Guangdong Medical University, Dongguan 523808, China

## Abstract

**Background:**

The relationship between vitamin D level and NAFLD has not been investigated in children and adolescents. We performed a meta-analysis of published observational studies to assess this association between vitamin D levels (measured as serum 25-hydroxy vitamin D [25(OH)D]) and NAFLD in this age group.

**Methods:**

Relevant studies conducted before May 20, 2018, were identified from the following electronic databases: PubMed, the Cochrane Library, Embase, and the Chinese CNKI databases. The quality of the included studies was evaluated using the Newcastle Ottawa Scale, and associations between vitamin D levels and NAFLD were estimated using standardised mean differences (SMD) and 95% confidence interval (CI). Subgroup and sensitivity analysis were used to identify sources of heterogeneity, and publication bias was evaluated using funnel plots.

**Results:**

Eight articles were included in this meta-analysis. A significant difference was observed between low 25(OH)D levels and NAFLD in children and adolescents (SMD = -0.59, 95%CI = -0.98, -0.20,* P *<* * 0.01). Subgroup analysis revealed no differences in the study type, geographic location, BMI, and age subgroups.

**Conclusions:**

Low vitamin D levels were associated with NAFLD in children and adolescents.

## 1. Introduction

Nonalcoholic fatty liver disease (NAFLD) is characterised by diffuse hepatocellular bullae and fatty changes detected in individuals with no evidence of excessive alcohol consumption [1]. NAFLD is a progressive disorder that ranges from simple steatosis to nonalcoholic steatohepatitis (NASH), and even to fibrosis and cirrhosis that can further progress to liver failure or hepatocellular carcinoma. The global prevalence of NAFLD in the general population is estimated at 20%–30% in western countries and 5%–18% in Asia, and it continues to grow [2, 3]. Over the past two decades, the epidemic of overweight and obesity has made the development of NAFLD, the leading cause of chronic liver disease worldwide. However, the risk factors of NAFLD are unclear and difficult to assess with precision, owing to lack of simple, noninvasive diagnostic tests [2, 4].

Vitamin D plays a key role in calcium and phosphorus homeostasis and is implicated in many diseases, including cardiovascular disease, autoimmune disease, infectious disease, and common cancers [5–7]. The main storage form of vitamin D is 25-hydroxyvitamin D [25(OH)D], which is converted to a biologically active form 1,25-dihydroxy vitamin D [1,25(OH)_2_D] in kidneys [8]. Vitamin D insufficiency and deficiency are also worldwide concerns, but they are no longer as extreme as in the past. Many studies have proposed that low vitamin D levels are strongly associated with features of metabolic syndrome [9]. Animal model studies have shown that vitamin D deficiency exacerbates NAFLD histology [10, 11]. Low vitamin D levels are also associated with liver steatosis, necroinflammation, and fibrosis in adult patients with NAFLD [12].

A link between low vitamin D levels and NAFLD has also been reported in a meta-analysis in adults, but data evaluated vitamin D status and NAFLD are sparse in pediatric population [13, 14]. Despite the similar characteristics of NAFLD shared with adult and adolescent patients, differences in histology are evident; for example, severe obesity adolescents may have a more aggressive NAFLD course with high risks for liver-related morbidities and mortality, and adolescent and adult severe obesity populations may have coinciding but changed NAFLD etiologies or risk factors [15, 16]. In recent years, research concerning the association between vitamin D levels and NAFLD could potentially lead to better insight into the pathogenesis of NAFLD in children and adolescents. And a recent study found no direct relationship between vitamin D deficiency and hepatosteatosis [17]. At present, data on an association between vitamin D levels and NAFLD in children and adolescents are lacking because of the difficulty of obtaining liver tissue specimens for determination of circulating vitamin D levels. Thus, the role of vitamin D in children and adolescents with NAFLD remains controversial. To the best of our knowledge, no meta-analysis data has yet examined the possible correlation between vitamin D levels and NAFLD in children and adolescents. Thus we conducted a meta-analysis to investigate the relationship between vitamin D deficiency and NAFLD in children and adolescents.

## 2. Materials and Methods 

### 2.1. Search Strategy

The methodology was based on the Preferred Reporting Items for Systematic Reviews and Meta Analyses (PRISMA) and the Cochrane Collaboration guidelines. We conducted systematic computerised searches in the PubMed, the Cochrane Library, and Embase databases, as well as in the China National Knowledge Infrastructure, to detect all printed articles on the association between vitamin D and NAFLD in children and adolescents. We used combinations of the following key words: (Vitamin D or 25-hydroxyvitamin D or 1,25-dihydroxy vitamin D or cholecalciferol or 25-hydroxyvitamin D_2_ or 25(OH)D or 25-hydroxycholecalciferol or 1,25(OH)_2_D or calcifediol) and (fatty liver or nonalcoholic fatty liver disease or NAFLD or hepatic steatosis or NASH or nonalcoholic steatohepatitis) and (children or adolescent). No language limitations were imposed. The final search was updated on May 20, 2018. Additional studies were manually identified from original studies or review articles that focused on this topic.

### 2.2. Inclusion and Exclusion Criteria

An article was selected if the study involved the association between vitamin D and children and adolescents with NAFLD or NASH, where NAFLD or NASH was diagnosed by histology or suggestive imaging features (ultrasound, computed tomography, magnetic resonance imaging, ultra-performance liquid chromatography tandem mass spectrometry), and/or suspected NAFLD was diagnosed by elevated ALT levels. The selection was not limited by design or language.

We further restricted the conditions to exclude animal studies, review articles, and studies in which the participants were adults. Studies were also excluded if they had liver injury participants induced by infections (hepatitis B virus or hepatitis C virus), alcohol, drugs, total parenteral nutrition, or hereditary causes. After scanning the articles, those with inadequate data or those only including NAFLD or NASH individuals without controls were excluded. After removing duplicates, the titles and abstracts of the articles were skimmed and irrelevant articles were excluded. At last, the full text of the enrolled articles was explored deeply to ensure selection only of relevant articles. To capture any additional relevant studies, the reference lists of all reviews and relevant articles were screened as well.

### 2.3. Data Extraction

Following the inclusion and exclusion criteria, data were extracted using a standardised extraction form by two investigators who screened, reviewed, and extracted data each paper independently. Disagreements were resolved by discussion and consensus. Using Cohen's kappa statistics (*κ*) [18], the overall agreement rate prior to correcting discrepancies was 0.85. Standardised abstraction sheets were used to record data from individual studies. Data retrieved from the studies included the first author's name, publication year, country of origin, sample size, study design, participant characteristics (age, gender, ethnicity, and body mass index), method of diagnosis of NAFLD and controls, and techniques for measuring serum 25(OH)D. If the published articles had inadequate information, attempts were made to contact the corresponding authors to acquire missing data. All data were double checked by another investigator.

### 2.4. Quality Assessment

This meta-analysis adopted the Newcastle Ottawa Scale (NOS), as suggested by the Cochrane Nonrandomised Studies Methods Working Group for assessment of case-control and cross-sectional studies. The NOS includes eight items under three categories: selection (four items, one star each), comparability (one item, up to two stars), and outcome (three items, one star each). A “star” represents a “high-quality” choice of an individual study. Given the variability in quality of the case-control and cross-sectional studies found in our initial literature search, we considered studies to be of high quality if they attained a score of six or more [19].

### 2.5. Statistical Analysis

We conducted the meta-analysis using STATA 12.0 (Stata Corporation, College Station, TX, USA). For studies that reported mean and standard deviation (SD) of serum 25(OH)D levels for NAFLD and controls, we combined the standardised mean differences (SMD), using Hedge's adjusted g to correct for small sample bias in a random effects model. Data from papers which did not present the mean and SD, values of median (m), range (a and b represent low and high end of range), Q_75_ (upper four quartile), and Q_25_ (lower four quartile) were converted into mean and SD based on the formulas shown in Table 1, and papers which involved two NAFLD or control groups were merged based on the following formulas [20, 21]:

(1) x−=N1M1+N2M2N1+N2,SD=N1−1SD12+N2−1SD22+N1N2/N1+N2M12+M22−2M1M2N1+N2−1.

Heterogeneity was assessed using the Cochran Q-statistic and I^2^-statistics (derived from Cochran's Q-statistic). For the Q statistic and I^2^-statistics,* P *< 0.10 and I^2^ > 50% were considered statistically significant for heterogeneity. Subgroup analysis was conducted to evaluate the effect of a potential factor on the association as a cause of heterogeneity such as stratification of the study type, geographic location, body mass index (BMI), and age [22]. The stability of the outcomes was assessed using sensitivity analysis by sequentially omitting each individual study using the “metaninf” STATA command. We assessed publication bias through visual inspection of funnel plot asymmetry, and asymmetry was tested by Egger's linear regression analysis.

## 3. Results

### 3.1. Study Inclusion and Characteristics

**Figure 1:**
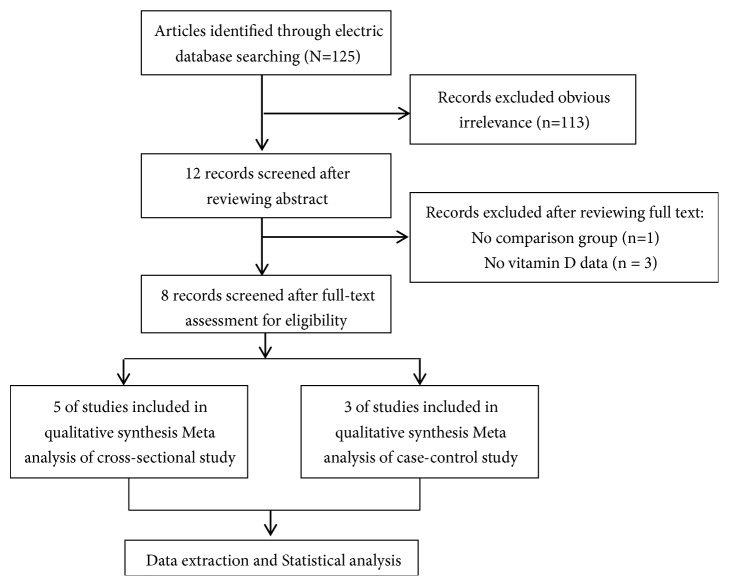
Literature screening flow chart.

A total of 125 potentially relevant articles were retrieved through the computer database and manual search. Of these, 113 were excluded after the titles and abstracts were reviewed because of a clear lack of relevance, and four were excluded after the full texts were reviewed because of a lack of control groups and vitamin D data. Ultimately, eight studies that met our inclusion criteria were included in the meta-analysis. All included studies were published in English. The complete search strategy is presented in Figure 1.

The eight included studies were three case-control studies [23–25] and five cross-sectional studies [17, 26–29]. The characteristics of all studies on the association between vitamin D and NAFLD in children and adolescents are presented in Tables 2 and 3.

The total sample size of the studies used to analyse the continuous vitamin D data for the NAFLD condition was 2052 participants (524 NAFLD cases and 1528 controls). The results of six studies showed that the level of vitamin D was lower in the NAFLD group than in the control group (*P *< 0.05). We merged the groups of Chang et al. [23] in our meta-analysis, because they divided NAFLD into three groups.

### 3.2. Literature Quality Evaluation Results

The quality evaluation results showed that diagnosis of NAFLD was clear, the vitamin D detection method was reasonable, the comparisons between groups were good, and the data were complete. Following the NOS guidelines [30], the total score of all the included studies was 6 “stars” and above, and the overall quality of the studies was good. The results are shown in Table 4.

### 3.3. Continuous Data of Vitamin D Levels and NAFLD in Children and Adolescents

**Figure 2:**
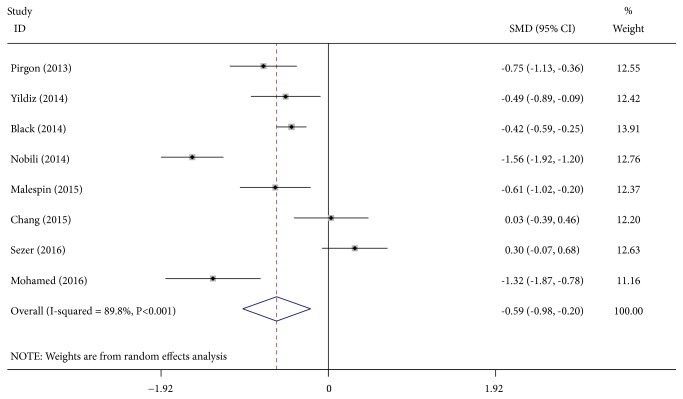
Forest plot of the studies comparing the association between vitamin D levels and children and adolescents with NAFLD by meta-analysis with the random effects analysis.

Heterogeneity tests indicated that a large heterogeneity existed between the eight studies included in the meta-analysis (I^2^ = 89.8%,* P *< * *0.001) (Figure 2), and a random effects model was used to merge the statistics and draw the forest plot (Figure 2). The forest plot showed that the diamonds are almost located on the left side of the midline, indicating that the levels of 25(OH)D were significantly lower in the patients with NAFLD than in the controls (SMD = -0.59, 95% CI = -0.98, -0.20,* P *= 0.003) (I^2^ = 89.8%,* P* <  * *0.001).

### 3.4. Subgroup Analysis

Subgroup analyses showed that a few differences were noted between the SMD and 95% CI in the subgroups (Table 5). However, it was not a significant change (case-control: SMD = -0.42, 95% CI = -0.67, -0.16; cross-sectional: SMD = -0.79, 95% CI = -1.67, 0.09) (Western: SMD = -6.04, 95% CI = -9.89, -2.18; Eastern: SMD = -6.30, 95% CI = -12.03, -0.57) (obese: SMD = -6.10, 95% CI = -12.84, 0.64; non-obese: SMD = -6.74, 95% CI = -13.10, -0.38) and (adolescents: SMD = -0.606, 95% CI = -1.129, -0.082; children: SMD = -0.575, 95% CI = -1.285, -0.135). The subgroup analysis suggested that a difference existed in the vitamin D levels between the NAFLD group and the control group.

### 3.5. Sensitivity Analysis

**Figure 3:**
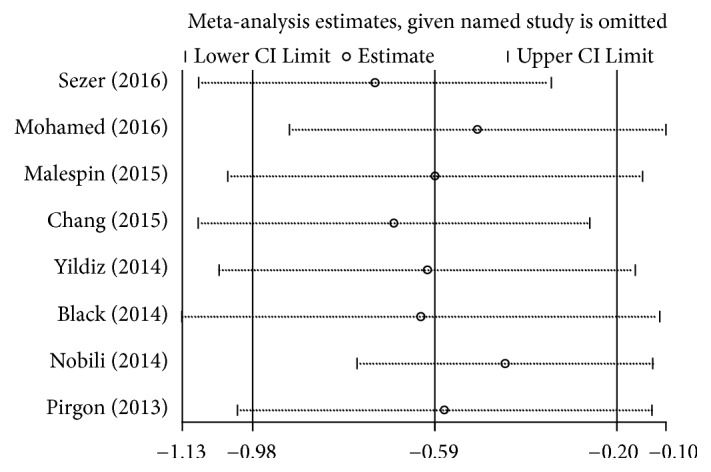
The sensitivity analysis for the association between vitamin D levels and NAFLD in children and adolescents by the random effects analysis.

As shown in Figure 3, the omission of the studies by Mohamed et al. [26] and Black et al. [28] resulted in a greater change in the estimated values than did omission of the other studies. However, significant relationships were noted between the low levels of vitamin D and children with NAFLD in all situations. After analysis, all the evidence from funnel plots and Egger's test showed that no publication bias existed in the meta-analysis (data not shown).

## 4. Discussion

Eight articles—five cross-sectional and three case-control studies—were included in this meta-analysis. The statistical analysis showed that levels of 25(OH)D were significantly lower in children and adolescents with NAFLD than in the controls (SMD = -0.59, 95% CI = -0.98, -0.20,* P *= 0.003). Further subgroup analysis based on study type, geographic location, BMI, and age resulted in no essential changes. A forest plot, which focused on the effect size, showed that vitamin D deficiency was 0.54 times less likely in children and adolescents with NAFLD than in the controls. Our findings showed that vitamin D levels were indeed lower in children and adolescents with NAFLD than in control without NAFLD.

Now NAFLD has become the most common chronic liver disease among children and adolescents. However, the screening and diagnostic methods used for paediatric NAFLD are not well defined [31, 32]. Many studies suggesting that insulin resistance (IR) may strongly be associated with NAFLD, but the mechanisms underlying this association remain uncertain [33, 34]. Kitade et al. [35] found that excessive hepatic lipid accumulation induced the activation of macrophages and Kupffer cells, leading to an exaggeration of IR, as well as hepatic inflammation and fibrogenesis. Vitamin D is produced by human skin in response to the sun. Vitamin D also has various functions in addition to maintaining calcium and bone homeostasis, involving cellular proliferation, anti-inflammatory and immune-modulatory functions, and differentiation. However, the various functions of vitamin D are not limited to these functions and may extend to the protection of insulin secretion and insulin sensitivity [6, 36]. Evidence now suggests that vitamin D deficiency contributes to the progression of both IR and NAFLD and aggravates NAFLD via TLR-activation, perhaps by way of endotoxin exposure [10, 37]. Luger et al. [38] found that increasing serum indicators of IR and vitamin D deficiency are clinically relevant predictors of fibrosis.

To our knowledge, this is the first meta-analysis to investigate the association of vitamin D levels with children and adolescents with NAFLD. The method of our study had no language restrictions, thus adding strength to our study. However, the present meta-analysis had several limitations. First, the study type of the included papers was mostly observational study, which cannot easily explain the causality. Second, the diagnostic methods differed among the included papers, and this would be expected to affect the results. Third, the subgroup analysis was performed on only the study type, geographic location, and BMI [13, 14]. Some researchers have hypothesised that vitamin D deficiency may be a confounding factor for obesity associated with IR in NAFLD [39]. The results of the previous studies were consistent with our findings, despite differences in the study populations. Our meta-analysis, which included case-control and cross-sectional studies, could not readily identify a causal relationship between vitamin D and NAFLD. The potential use of vitamin D in the treatment of NAFLD has become more intriguing with the introduction of a preclinical model of steatosis, but the present study still lacks evidence from clinical trials using vitamin D supplementation in children with NAFLD. In summary, further prospective clinical studies and randomised controlled trials are needed to provide causal evidence to support the results of observational and animal studies.

## 5. Conclusion

In conclusion, our results indicated that vitamin D deficiency prevails in children and adolescents with NAFLD and vitamin D may be associated with the progression and severity of NAFLD. The relationship was analysed by subgroups, but no essential differences were noted between the subgroups. Therefore, future studies should focus on new mechanisms that can link vitamin D deficiency and children and adolescents with NAFLD. More clinical trials using vitamin D supplementation are also needed in children and adolescents with NAFLD.

## Figures and Tables

**Table 1:** The best estimating formula for an unknown distribution.

Sample size (N)	Mean ($\overset{-}{x}$)	Standard deviation (SD)
N ≤ 15	$\frac{a+2m+b}{4}$	$\sqrt{\frac{1}{12}\left[\frac{{\left(a-2m+b\right)}^{2}}{4}+{\left(b-a\right)}^{2}\right]}$
15 < N ≤ 25	Median	$\frac{b-a}{4}$
25 < N ≤ 70	Median	$\frac{b-a}{4}$ OR $\frac{Q_{75}-Q_{25}}{1.35}$
N >70	Median	$\frac{b-a}{6}$

m: values of median; a: low end of range; b: high end of range; Q_75_: upper four quartile; Q_25_: lower four quartile.

**Table 2:** Main characteristics of studies on continuous outcomes of vitamin D levels in NAFLD and controls, chronologically ordered.

Study	Year	Control			NAFLD			*P*-value^*∗*^
		Age (mean ± SD)	No. (Female/male)	25(OH)D (ng/ml) (mean ± SD)	Age (mean ± SD)	No. (Female/male)	25(OH)D (ng/ml) (mean ± SD)	
Sezer et al.[17]	2016	12.60±3.10	53(38/15)	14.60±7.00	12.90±2.20	58(26/32)	16.60±6.20	0.113
Mohamed et al. [26]	2016	10.60±3.10	23(14/9)	41.98±14.52	11.13±2.70	47(28/19)	20.89±16.56	<0.001
Malespin et al. [27]	2015	12.90±2.90	382(193/189)	20.80±7.10	13.00±2.40	25(6/19)	16.50±6.40	0.024
Chang et al. [23]	2015	8.70±3.87	32(NR)	17.70±5.05	11.46±2.53	62(NR)	17.90±6.84	0.963
Yildiz et al. [24]	2014	11.00±2.80	43(24/19)	16.40±9.19	11.90±2.80	58(22/36)	12.60±6.52	0.005
Black et al.[28]	2014	17	838(381/457)	30.85±9.62	17	156(96/60)	26.84±8.81	<0.001
Nobili et al. [29]	2013	12.24±7.23	85(32/53)	29.04±5.81	13.00±2.96	73(NR)	19.30±6.70	<0.001
Pirgon et al. [25]	2013	12.48±1.60	72(NR)	43.96±19.97	12.80±0.80	45(NR)	29.50±18.40	<0.05

^*∗*^The levels of 25(OH)D were compared between NAFLD and control groups; SD: standard deviation; NR: not reported.

**Table 3:** Main characteristics of studies on the association between vitamin D and NAFLD, ordered by year of publication.

Study (authors-year)	Country	Race/ ethnicity	Study type	Health status (Case)	setting	Method of NAFLD ascertainment	BMI in NAFLD (mean±SD)	BMI in controls (mean±SD)
Sezer et al. (2016) [17]	Turkey	Turkish	Cross-sectional	Hepatosteatosis	outpatient	ultrasound	28.7±4.3	27.3±3.3
Mohamed et al. (2016) [26]	Egypt	Egyptian	Cross-sectional	NAFLD	outpatient	ultrasound	NR	NR
Malespin et al. (2015) [27]	USA	Chinese	Cross-sectional	Suspected NAFLD	NR	Elevated ALT	NR	NR
Chang et al. (2015) [23]	Korea	Korean	Case control	SS and NASH	outpatient	UPLC- MS/MS	25.89±4.13	23.7±2.63
Yildiz et al. (2014) [24]	Turkey	Turkish	Case control	Hepatosteatosis	Pediatrics clinic	ultrasound	30.9±3.9	29.3±4.4
Black et al. (2014) [28]	Australia	Caucasian Non-Caucasian	Cross-sectional	NAFLD	General population	ultrasound	27.0±7.4	22.0±3.0
Nobili et al. (2014) [29]	Italy	Caucasian	Cross-sectional	NAFLD	Liver biopsy	ultrasound	31.3±4.37	NR
Pirgon et al. (2013) [25]	Turkey	Turkish	Case control	NAFLD	Inpatient	ultrasound	28.7±4.7	28.4±3.6

SS: simple steatosis; NASH: nonalcoholic steatohepatitis; NAFLD: nonalcoholic fatty liver disease; BMI was measured in kg/m^2^; ALT: alanine aminotransferase; UPLC-MS/MS: ultra-performance liquid chromatography tandem mass spectrometry.

**Table 4:** Newcastle Ottawa Scale (NOS) assessment of the quality of the case-control and cross-sectional studies.

Study	Selection			Comparability			Exposure		Total scores
	Case definition adequate	Representativeness of the cases	Selection of controls	definition of Controls	Comparability based on design or analysis	Ascertainment of exposure	Same method of ascertainment for cases and controls	Non-response rate	
Sezer et al. [17]	★	★	★	★		★	★		6
Mohamed et al. [26]	★	★	★	★	★	★	★		8
Malespin et al. [27]	★	★		★	★	★	★		6
Chang et al. [23]	★	★	★	★		★	★		6
Yildiz et al. [24]	★	★	★	★	★★	★	★		8
Black et al. [28]	★	★	★	★	★	★	★		7
Nobili et al. [29]	★	★		★	★	★	★		6
Pirgon et al. [25]	★	★		★	★★		★		6

**Table 5:** Subgroup analysis of studies comparing the association between vitamin D levels and children and adolescents with NAFLD.

Subgroups	No. (NAFLD/Control)	Pooled SMD (95% CI)	*P*_*s*_	I^2^ (%)	*P*
25(OH)D (ng/ml)					
Overall	2052 (524/1528)	-0.593 (-0.983, -0.204)	*P *= 0.003	89.8%	*P*<0.001
Study type					
Cross-sectional	746 (203/543)	-0.793 (-1.673, 0.088)	*P *= 0.078	94.5%	*P*<0.001
Case-control	1306 (321/985)	-0.418 (-0.674, -0.162)	*P* = 0.001	58.2%	*P* = 0.066
Geographic location					
Western	1559 (254/1305)	-0.856 (-1.558, -0.154)	*P* = 0.017	93.7%	*P*<0.001
Eastern	493 (270/223)	-0.428 (-0.955, 0.098)	*P *= 0.111	87.3%	*P*<0.001
BMI (kg/m^2^)					
Obese	581 (296/285)	-0.495 (-1.160, 0.170)	*P* = 0.144	93.2%	*P*<0.001
Non-obese	1064 (203/861)	-0.834 (-1.715, 0.046)	*P *= 0.063	89.5%	*P* = 0.002
Else	407 (25/382)	-0.609 (-1.016, -0.202)	*P* = 0.003	NR	NR
Age (years)					
Children	265(167/98)	-0.575 (-1.285,0.135)	*P* = 0.112	86.4%	*P *= 0.001
Adolescents	1787(357/1430)	-0.606 (-1.129, -0.082)	*P* = 0.023	92.6%	*P*<0.001

*Ps* denotes *P* value for heterogeneity based on Q test; *P* denotes *P* value for statistical significance based on Z test.

## Data Availability

The data used to support the findings of this study are included within the article.

## References

[1] Sun C., Fan J. G., Qiao L. (2015). Potential epigenetic mechanism in non-alcoholic fatty liver disease. *International Journal of Molecular Sciences*.

[2] Satapathy S. K., Sanyal A. J. (2015). Epidemiology and natural history of nonalcoholic fatty liver disease. *Seminars in Liver Disease*.

[3] Pawar S. V., Zanwar V. G., Choksey A. S. (2016). Most overweight and obese Indian children have nonalcoholic fatty liver disease. *Annals of Hepatology*.

[4] Neuman M. G, Nanau R. M, Cohen L. B. (2015). Nonmedicinal interventions in nonalcoholic fatty liver disease. *Canadian Journal of Gastroenterology and Hepatology*.

[5] Ponchon G., Deluca H. F. (1969). The role of the liver in the metabolism of vitamin D. *The Journal of Clinical Investigation*.

[6] Torun E., Gönüllü E., Özgen İ. T., Cindemir E., Öktem F. (2013). Vitamin D deficiency and insufficiency in obese children and adolescents and its relationship with insulin resistance. *International Journal of Endocrinology*.

[7] Nalbant A., Vatan M. B., Varım P., Varım C., Kaya T., Tamer A. (2017). Does vitamin D deficiency effect heart rate variability in low cardiovascular risk population?. *Open Access Macedonian Journal of Medical Sciences*.

[8] Borel P., Caillaud D., Cano N. J. (2015). Vitamin D bioavailability: state of the art. *Critical Reviews in Food Science and Nutrition*.

[9] Gulseth H. L., Gjelstad I. M., Birkeland K. I., Drevon C. A. (2013). Vitamin D and the metabolic syndrome. *Current Vascular Pharmacology*.

[10] Roth C. L., Elfers C. T., Figlewicz D. P. (2012). Vitamin D deficiency in obese rats exacerbates nonalcoholic fatty liver disease and increases hepatic resistin and toll-like receptor activation. *Hepatology*.

[11] Kang E. J., Lee J. E., An S. M. (2015). The effects of vitamin D3 on lipogenesis in the liver and adipose tissue of pregnant rats. *International Journal of Molecular Medicine*.

[12] Lu Z., Pan X., Hu Y. (2015). Serum vitamin D levels are inversely related with non-alcoholic fatty liver disease independent of visceral obesity in Chinese postmenopausal women. *Clinical and Experimental Pharmacology and Physiology*.

[13] Eliades M., Spyrou E., Agrawal N. (2013). Meta-analysis: vitamin D and non-alcoholic fatty liver disease. *Alimentary Pharmacology & Therapeutics*.

[14] Wang X., Li W., Zhang Y., Yang Y., Qin G. (2015). Association between vitamin D and non-alcoholic fatty liver disease/non-alcoholic steatohepatitis: results from a meta-analysis. *International Journal of Clinical and Experimental Medicine*.

[15] Tsai P. (2015). The Vitamin D Controversy. *Journal of Pediatric Gastroenterology and Nutrition*.

[16] Hourigan S. K., Abrams S., Yates K. (2015). Relation between vitamin D status and nonalcoholic fatty liver disease in children. *Journal of Pediatric Gastroenterology and Nutrition*.

[17] Sezer O. B., Bulus D., Hizli S., Andlran N., Yllmaz D., Ramadan S. U. (2016). Low 25-hydroxyvitamin D level is not an independent risk factor for hepatosteatosis in obese children. *Journal of Pediatric Endocrinology and Metabolism*.

[18] Cohen J. (1968). Weighted kappa: nominal scale agreement provision for scaled disagreement or partial credit. *Psychological Bulletin*.

[19] Puigvehí M., Hernández J., Broquetas T. (2016). Diagnostic accuracy of the enhanced liver fibrosis (ELF®) score using HCV-infected serum samples cryopreserved for up to 25 years. *PLoS ONE*.

[20] Hozo S. P., Djulbegovic B., Hozo I. (2005). Estimating the mean and variance from the median, range, and the size of a sample. *BMC Medical Research Methodology*.

[21] Zhang T. S., Zhong W. Z., Li B. (2014). Practical evidence - based medicine methodology. *Practical Evidence - Based Medicine Methodology*.

[22] Higgins J., Green S. (2008). *Cochrane Handbook for Systematic Reviews of Interventions*.

[23] Chang E. J., Yi D. Y. (2015). Vitamin D status and bone mineral density in obese children with nonalcoholic fatty liver disease. *Gastroenterology & Hepatology*.

[24] Yildiz I., Erol O. B., Toprak S. (2014). Role of vitamin D in children with hepatosteatosis. *Journal of Pediatric Gastroenterology and Nutrition*.

[25] Pirgon O., Cekmez F., Bilgin H., Eren E., Dundar B. (2013). Low 25-hydroxyvitamin D level is associated with insulin sensitivity in obese adolescents with non-alcoholic fatty liver disease. *Obesity Research & Clinical Practice*.

[26] Ahmed A. M., Abdel Ghany M., Abdel Hakeem G. L. (2016). Assessment of vitamin D status in a group of Egyptian children with non alcoholic fatty liver disease (multicenter study). *Nutrition & Metabolism*.

[27] Malespin M., Sleesman B., Lau A., Wong S. S., Cotler S. J. (2015). Prevalence and correlates of suspected nonalcoholic fatty liver disease in Chinese American children. *Journal of Clinical Gastroenterology*.

[28] Black L. J., Jacoby P., Ping-Delfos W. C. S. (2014). Low serum 25-hydroxyvitamin D concentrations associate with non-alcoholic fatty liver disease in adolescents independent of adiposity. *Journal of Gastroenterology and Hepatology*.

[29] Nobili V., Giorgio V., Liccardo D. (2014). Vitamin D levels and liver histological alterations in children with nonalcoholic fatty liver disease. *European Journal of Endocrinology*.

[30] Wells G., Shea B., OConnell D. The newcastle-ottawa scale (NOS) for assessing the quality if nonrandomized studies in meta-analyses. http://www.ohri.ca/programs/clinical_epidemiology/oxford.htm.

[31] Selvakumar P. K., Kabbany M. N., Nobili V., Alkhouri N. (2017). Nonalcoholic fatty liver disease in children: hepatic and extrahepatic complications. *Pediatric Clinics of North America*.

[32] Uppal V., Mansoor S., Furuya K. N. (2016). Pediatric non-alcoholic fatty liver disease. *Current Fungal Infection Reports*.

[33] Asrih M., Jornayvaz F. R. (2015). Metabolic syndrome and nonalcoholic fatty liver disease: is insulin resistance the link?. *Molecular and Cellular Endocrinology*.

[34] Jelenik T., Kaul K., Séquaris G. (2017). Mechanisms of insulin resistance in primary and secondary Non-alcoholic fatty liver. *Diabetes*.

[35] Kitade H., Chen G., Ni Y., Ota T. (2017). Nonalcoholic fatty liver disease and insulin resistance: new insights and potential new treatments. *Nutrients*.

[36] Alvarez J. A., Ashraf A. (2010). Role of vitamin D in insulin secretion and insulin sensitivity for glucose homeostasis. *International Journal of Endocrinology*.

[37] Dongiovanni P., Lanti C., Riso P., Valenti L. (2016). Nutritional therapy for nonalcoholic fatty liver disease. *The Journal of Nutritional Biochemistry*.

[38] Luger M., Kruschitz R., Kienbacher C. (2016). Prevalence of liver fibrosis and its association with non-invasive fibrosis and metabolic markers in morbidly obese patients with vitamin D deficiency. *Obesity Surgery*.

[39] Catena C., Cosma C., Camozzi V. (2013). Non-alcoholic fatty liver disease is not associated with vitamin D deficiency in essential hypertension. *High Blood Pressure & Cardiovascular Prevention*.

